# Editorial: Neurocinematics: how the brain perceives audiovisuals

**DOI:** 10.3389/fnins.2025.1719407

**Published:** 2025-10-29

**Authors:** Celia Andreu-Sánchez, Miguel Ángel Martín-Pascual, José María Delgado-García

**Affiliations:** ^1^Neuro-Com Research Group, Universitat Autònoma de Barcelona, Barcelona, Spain; ^2^Research and Development, Radio Televisión Española Instituto, Barcelona, Spain; ^3^Division of Neurosciences, Pablo de Olavide University, Seville, Spain

**Keywords:** neurocinematics, visual perception, cognitive neuroscience, media, audiovisuals, neurophysiology

Understanding how the brain perceives audiovisuals seems crucial in the current screen era (Marciano et al., 2021). This type of research provides quantitative and reliable tools for designing media content based on neural responses rather than subjective reports. Identifying patterns that link brain activity with audiovisual characteristics, whether format or content-related, can offer profound insights into brain function in response to different stimuli.

Recent studies have shown that cognitive deficits can become apparent when perceiving audiovisuals, highlighting the potential for developing non-invasive tools to address these deficits (Chu et al., 2023; Li et al., 2025, 2024). Despite significant advancements, there remain gaps in our understanding of how media content impacts brain activity and, consequently, behavior and cognition. This underscores the need for further investigation into the neural mechanisms underlying audiovisual perception.

This Research Topic aimed to expand our knowledge of neurocinematics, an interdisciplinary approach to understanding audiovisual perception through the brain activity of spectators (Hasson et al., 2004, 2008). The primary objectives included exploring how the brain processes audiovisuals, identifying neural patterns associated with different types of media content, and understanding the cognitive and physiological responses elicited by audiovisual stimuli.

The articles contributing to this Research Topic address this critical goal by leveraging advanced neuroscientific and psychophysiological methodologies to explore the complexities of media perception, spanning fundamental film grammar, emotional processing in ecologically valid settings, and the emergence of immersive media formats.

## Decoding cinematic grammar and temporal integration

A key area of investigation focuses on how the brain processes the most fundamental element of film structure: the editing cut. Sanz-Aznar explores whether the cinematographic cut functions as an articulation axis between adjacent shots. Through frequency domain analysis (Event-Related Desynchronization/Synchronization,ERD/ERS) of electroencephalographic (EEG) recordings, the study confirmed that continuity cuts elicit neural response patterns in theta-band synchronization and delta-band desynchronization, which are associated with memory encoding, narrative segmentation, and meaning construction. These results support the hypothesis that the shot change is neurally processed as an articulatory mechanism within film structure.

Complementing this, research presented by Cancer et al. investigates the mechanisms underlying the effect of editing density (master shot, slow-paced, fast-paced) on the viewer's perception of time. The study found that neuromodulation of the supplementary motor area (SMA) affected duration estimates and action speed judgments, highlighting the importance of the SMA in modulating the perception of filmic time during viewing.

To better characterize the overall integration of complex stimuli, Xi et al. present a novel EEG microstate-based method to calculate time-frequency features of multi-stage audiovisual (AV) neural processing. This study's approach segments AV processing into multiple distinct sub-stages: six under attended conditions and four under unattended conditions. The resulting time-frequency domain features achieved high classification accuracy (up to 97.8%) in distinguishing attended vs. unattended AV processing, demonstrating the method's effectiveness in providing a high-resolution temporal window into the brain's information processing stages.

## Emotion, aesthetics, and predictive cognition

This Research Topic also approaches the influence of specific visual and aesthetic elements on emotional processing. Huttunen investigated the effects of lighting direction on the early emotional processing of faces. Measuring the Early Posterior Negativity (EPN), the study found that lighting styles that obscure or distort facial information (silhouette light, underlight, and toplight) elicited a statistically more negative EPN compared to neutral 45-degree light. This suggests that lighting design impacts the film experience at the subliminal level of emotional processing.

Extending to real-world media analysis, Martín-Pascual and Andreu-Sánchez used eye-tracking to examine the visual perception of war images in multi-window Spanish television news. They found that viewers dedicated the longest gaze duration and highest fixation count to the war imagery itself, compared to the journalist or explanatory texts. Notably, images depicting deceased individuals drew more time and fixations, potentially indicating the selective engagement of memory processes. Moreover, patterns of visual attention varied across political leaders, with Vladimir Putin attracting greater focus than Volodymyr Zelensky, a difference that was linked to stronger negative emotions.

Theoretically framing these aesthetic and narrative processes, Coëgnarts proposes integrating the Predictive Processing (PP) framework with cinematic aesthetics. PP posits that the mind acts as a predictive machine that minimizes error by anticipating sensory input. This theoretical application highlights how films deliberately engage the brain's inferential processes, proposing that image schemas function as mechanisms structuring sensory input in alignment with the brain's predictive logic, thereby enhancing comprehension and aesthetic pleasure.

## Navigating the challenges of immersive media

The complexities of translating traditional cinematic grammar to new formats, particularly Virtual Reality (VR), are explored by Zou et al. This study investigated how different editing techniques (unedited, hard cuts, dissolve transitions) in VR films affect narrative cognition. Findings show that unedited VR films promote superior emotional coherence and immersion, likely driven by sustained amygdala activation. However, this format also imposes a higher cognitive load. Conversely, edited films reduced cognitive load but resulted in fragmented attention and diminished emotional engagement. Dissolve transitions were found to be especially detrimental to viewer enjoyment. These results show that VR editing places specific neurocognitive demands, emphasizing the need for flexible strategies that prioritize smooth transitions to maintain emotional engagement.

## Embodiment, physiology, and inclusivity

Beyond the viewer's experience, Primett et al. explore the cinematographer's embodied experience during creation. Utilizing a neurophenomenological methodology combining physiological data (electrodermal activity, EDA, electrocardiogram, ECG), motion data, and micro-phenomenological interviews, the research investigates kinaesthetic empathy, highlighting how the cinematographer's embodied decision-making shapes and modulates emotional understanding throughout the filming process.

From another perspective, Kolesnikov et al. study how drone movements, human presence, and playback speed affect viewers' sense of movement, involvement, emotion, and time. Ascending trajectories provoked the highest levels of movement perception, aesthetic appreciation and emotional engagement. Results suggest drone cinematography can intensify embodied and emotional responses, supporting embodied simulation theory.

Addressing critical issues of accessibility, Yang et al. investigated the impact of film Audio Description (AD) style on the sense of presence in Chinese visually impaired audiences. They determined that subjective AD (which incorporates interpretation and emotional coloring) significantly enhanced key dimensions of presence—including engagement, spatial awareness, and ecological validity—compared to the more neutral, objective style.

Finally, Fusina et al. contribute to the understanding of individual differences in emotional processing by examining the heart-brain connection during ecological emotional film viewing. They focused on women with high (HD) and low (LD) traits of emotion dysregulation and found a significant positive correlation between heart rate (HR) and gamma band connectivity within the Ventral Attention Network (VAN) in the HD group, particularly during sadness and neutral clips. This consistent correlation suggests that sadness acts as a synchronizing agent coordinating cardiovascular and central cortical responses in this population.
